# Correction: Fluorescent metabolic labeling-based quick antibiotic susceptibility test for anaerobic bacteria

**DOI:** 10.1039/d2cb90037h

**Published:** 2022-10-03

**Authors:** Juan Gao, Juanxiu Qin, Chenling Ding, Yuan Gao, Junnan Guo, Min Li, Chaoyong Yang, Wei Wang

**Affiliations:** Institute of Molecular Medicine, Renji Hospital, Shanghai Jiao Tong University School of Medicine Shanghai 200127 China cyyang@xmu.edu.cn wwang@shsmu.edu.cn; Department of Laboratory Medicine, Renji Hospital, Shanghai Jiao Tong University School of Medicine Shanghai 200127 China ruth_limin@126.com; Department of Critical Care Medicine, Renji Hospital, Shanghai Jiao Tong University School of Medicine Shanghai 200127 China; The MOE Key Laboratory of Spectrochemical Analysis and Instrumentation, Key Laboratory for Chemical Biology of Fujian Province State Key Laboratory of Physical Chemistry of Solid Surfaces, Department of Chemical Biology, College of Chemistry and Chemical Engineering Xiamen University Xiamen 361005 China

## Abstract

Correction for ‘Fluorescent metabolic labeling-based quick antibiotic susceptibility test for anaerobic bacteria’ by Juan Gao *et al.*, *RSC Chem. Biol.*, 2022, https://doi.org/10.1039/d2cb00163b.

Two of the affiliations (b and c) were reported in the wrong order in the original article.

A corrected version of the affiliation list is shown here, in which the Department of Laboratory Medicine, Renji Hospital, Shanghai Jiao Tong University School of Medicine has changed from affiliation c to b, and the Department of Critical Care Medicine, Renji Hospital, Shanghai Jiao Tong University School of Medicine has changed from affiliation b to c.

The Royal Society of Chemistry apologises for these errors and any consequent inconvenience to authors and readers.

## Supplementary Material

